# Successful Resolution of Coronary Thrombus in a Child With Kawasaki Disease and Giant Coronary Artery Aneurysms Using Anticoagulation Therapy

**DOI:** 10.7759/cureus.96767

**Published:** 2025-11-13

**Authors:** Hamza Almasaeid, Abdel-Aziz Saleh, Haya Obaidy

**Affiliations:** 1 General Physician, Al Khalidi Hospital, Amman, JOR

**Keywords:** coronary artery thrombosis, echocardiogram detecting coronary artery aneurysm, heparin therapy, kawasaki disease (kd), . pediatric cardiology

## Abstract

Kawasaki disease (KD) is a systemic vasculitis that can lead to coronary artery aneurysms (CAAs), particularly in untreated or treatment-resistant cases. Giant aneurysms carry a significant risk for thrombosis and myocardial ischemia. We report the case of a four-year-old boy with KD and multiple giant CAAs who presented with a sub-therapeutic international normalized ratio (INR). Echocardiography revealed a large thrombus in the left anterior descending (LAD) artery. The patient was treated with continuous heparin infusion, warfarin, and aspirin. Serial echocardiography confirmed complete thrombus resolution within one month. This case underscores the importance of consistent anticoagulation monitoring and individualized therapy in children with giant CAAs due to KD. Heparin infusion, followed by tailored warfarin therapy, can achieve safe and effective thrombus resolution without the need for surgical intervention.

## Introduction

Kawasaki disease (KD) is an acute, self-limited febrile illness of childhood that primarily affects children under the age of five years. It is a systemic vasculitis of unknown etiology that targets medium-sized vessels, particularly the coronary arteries. If untreated, up to 25% of patients may develop coronary artery aneurysms (CAAs), which are associated with thrombosis, myocardial infarction, and sudden cardiac death [1,2]. Echocardiography and coronary computed tomography angiography (CTA) are essential tools for assessing aneurysm size and location in KD. We present a case of thrombus formation in a child with multiple giant aneurysms due to KD, successfully managed with anticoagulation.

## Case presentation

**Figure 1:**
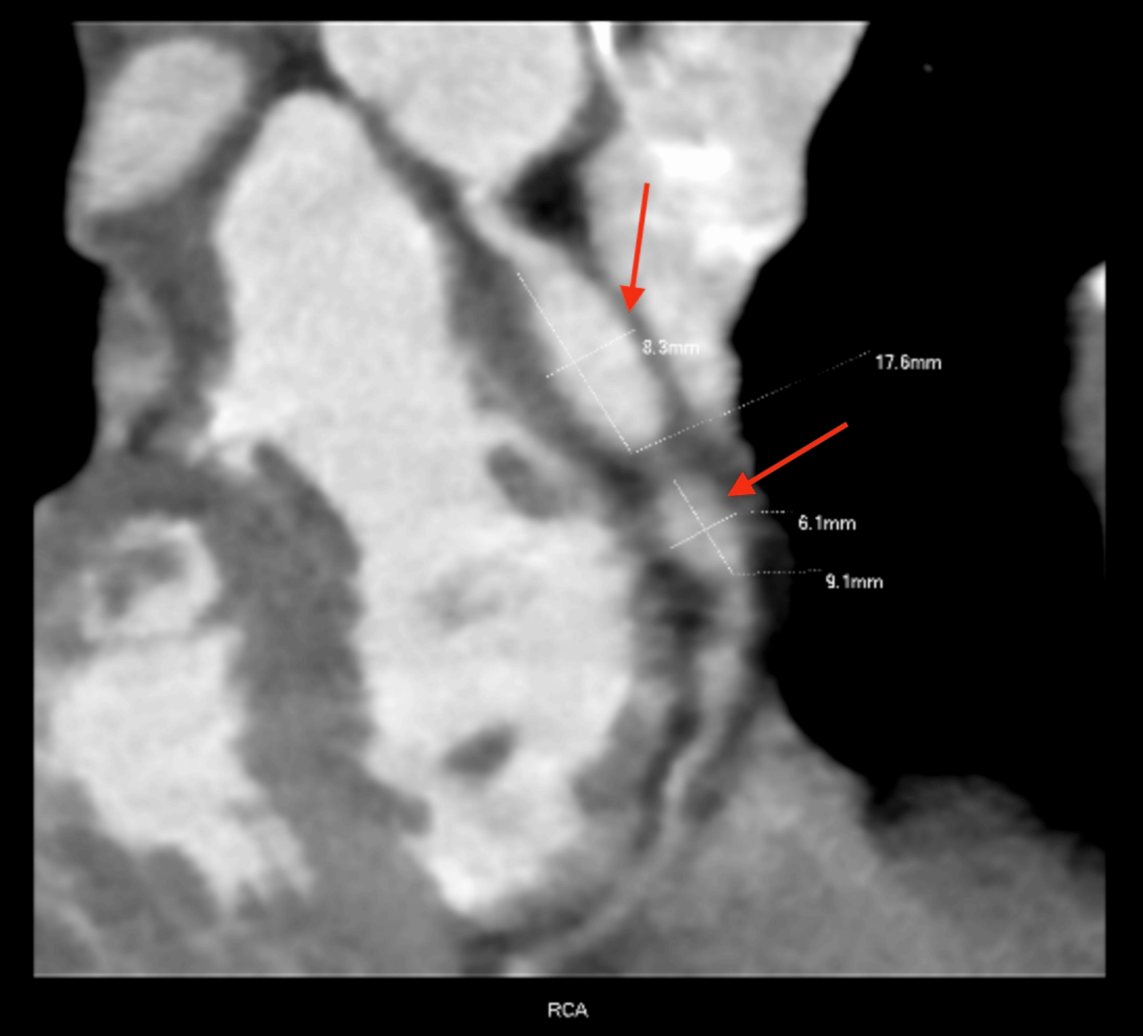
Coronary computed tomography angiography image of the patient The image is demonstrating multiple coronary artery aneurysms (red arrows).

A four-year-old boy with a prior diagnosis of KD at the age of 1.5 years was evaluated at a routine cardiology follow-up. Coronary computed tomography angiography (coronary CTA) had previously demonstrated multiple coronary artery aneurysms (Figure 1). In the right coronary artery (RCA), there was a giant fusiform aneurysm measuring 17.6 mm (Z = 44.9), a second aneurysm measuring 9.1 mm (Z = 18.5), and a third aneurysm measuring 5.6 mm (Z = 10.7). In the left anterior descending artery (LAD), a proximal fusiform aneurysm measured 22.4 mm (Z = 57.4). Body surface area (BSA) was calculated using Haycock’s formula (BSA = 0.678 m²), and coronary Z-scores were calculated using the regression equations of Dallaire and Dahdah (2011) [3].

**Figure 2:**
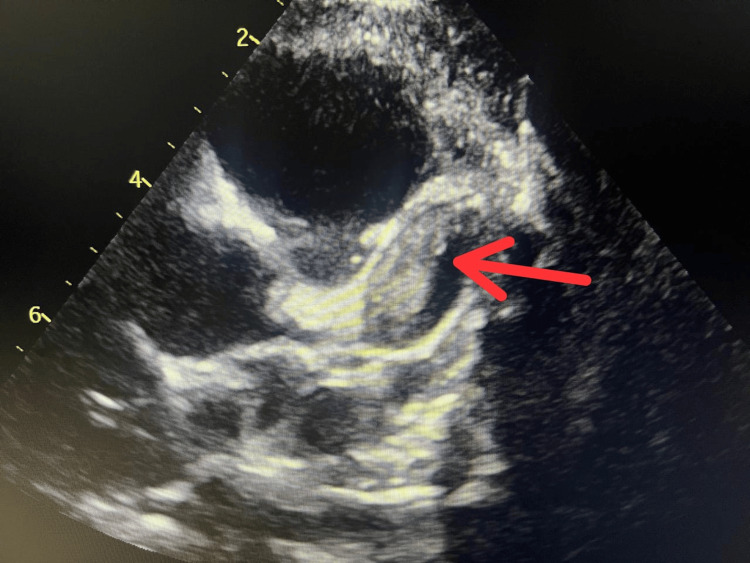
Echocardiographic image of the patient This echocardiographic image reveals a dilated left anterior descending (LAD) artery with a distinct echogenic structure within the lumen, consistent with thrombus.

Routine laboratory testing revealed a subtherapeutic international normalized ratio (INR) of 1.6. The patient was clinically well and asymptomatic, with no complaints of chest pain, dyspnea, syncope, or irritability. Transthoracic echocardiography performed at that visit demonstrated a dilated LAD containing a well-demarcated echogenic intraluminal mass consistent with thrombus (Figure 2). The patient was admitted to the coronary care unit and treated with a continuous unfractionated heparin infusion (target activated partial thromboplastin time > 60 seconds), concurrent aspirin, and initiation of warfarin. Anticoagulation was titrated according to serial activated partial thromboplastin time (aPTT) and INR measurements, with a targeted therapeutic INR range of 2.0-3.0.

**Figure 3:**
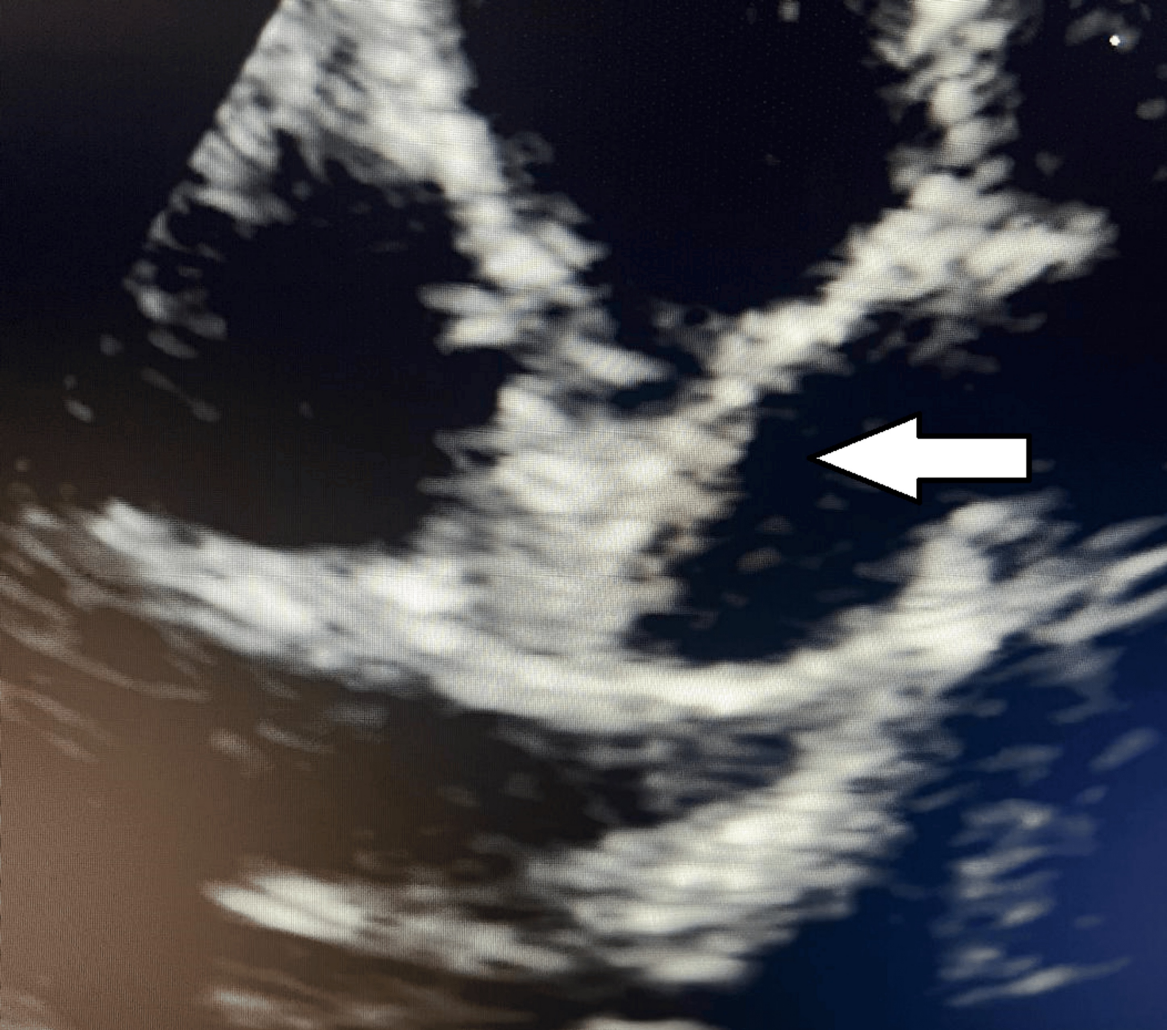
Follow-up echocardiography after anticoagulation therapy This follow-up image shows the previously dilated left anterior descending (LAD) artery without visible thrombus, indicating successful resolution of the thrombus following anticoagulation therapy.

Serial echocardiography documented progressive thrombus reduction, and no residual thrombus was visible after one month of therapy (Figure 3). The patient was discharged on oral warfarin and aspirin with a final INR of 2.26.

## Discussion

Kawasaki disease (KD) is the leading cause of acquired heart disease in children in developed countries, primarily leading to coronary artery aneurysms. First described in Japan in 1967, KD is a systemic inflammatory illness involving medium-sized vessels, particularly the coronary arteries. The cause remains uncertain, but evidence suggests that infectious or environmental factors may trigger the disease in genetically predisposed individuals [1,2]. The diagnosis of KD is clinical and is based on the following criteria: fever lasting at least five days plus four or more of the following: conjunctival injection, mucosal changes, cervical lymphadenopathy, polymorphous rash, and extremity changes. Incomplete or atypical KD, often seen in infants, is associated with delayed diagnosis and a higher risk of coronary complications [2,4].

Coronary artery aneurysms represent the most serious complication. These are classified as small (z-score ≥2.5 to <5, <5 mm), medium (z-score ≥5 to <10, 5-8 mm), or giant (z-score ≥10, >8 mm). Giant aneurysms have the highest risk of thrombosis, myocardial infarction, and sudden cardiac death, with reported thrombosis rates as high as 50% [5,6]. Risk factors for aneurysm formation include male sex, young age, delayed IVIG treatment, and persistent inflammation [4,7]. In our patient, CTA and echocardiography were instrumental in assessing the extent of aneurysmal disease and in detecting thrombus formation. Importantly, the thrombus developed despite the patient being asymptomatic, reinforcing the need for routine imaging and close anticoagulation monitoring.

Management of giant aneurysms typically involves dual or triple therapy, combining aspirin with systemic anticoagulation. Options include unfractionated heparin infusion, low-molecular-weight heparin, or oral anticoagulants such as warfarin. The use of heparin infusion enabled the rapid achievement of therapeutic anticoagulation in our patient, while serial echocardiography enabled noninvasive monitoring of thrombus resolution. This approach aided in avoiding the need for invasive angiography or surgical intervention. Our case supports published literature indicating that individualized anticoagulation strategies can reduce thrombus burden and improve outcomes in children with KD and giant aneurysms [7,8].

## Conclusions

This case highlights the critical importance of routine surveillance imaging and careful anticoagulation management in children with Kawasaki disease complicated by giant coronary artery aneurysms. Early detection of thrombus and prompt initiation of anticoagulation can lead to complete thrombus resolution and favorable outcomes without surgical intervention.
